# Chronic endometritis incidence in infertile women with and without polycystic ovary syndrome: a propensity score matched study

**DOI:** 10.1186/s12905-023-02759-5

**Published:** 2023-11-27

**Authors:** Jiayi Guo, Yajie Chang, Zhi Zeng, Huijun Liu, Xiaoyan Liang, Haitao Zeng, Jintao Peng

**Affiliations:** 1 Reproductive Medicine Center, The Sixth Affiliated Hospital, Sun Yat-sen University, Guangzhou, 510655 Guangdong China; 2 GuangDong Engineering Technology Research Center of Fertility Preservation, Guangzhou, 510655 Guangdong China; 3 Biomedical Innovation Center, The Sixth Affiliated Hospital, Sun Yat-sen University, Guangzhou, 510655 Guangdong China

**Keywords:** CD138, Chronic endometritis, Inflammation, Polycystic ovary syndrome, Phenotypes, Obesity, Propensity score matched study

## Abstract

**Background:**

Polycystic ovary syndrome (PCOS) is known to be associated with chronic low-grade inflammation and endometrial dysfunction. Chronic endometritis (CE) is a type of local inflammation that can contribute to endometrial dysfunction in infertile women. Some clinicians recommend screening for CE in women at high risk, such as those with endometrial polyps. However, it is still uncertain whether there is a relationship between PCOS and CE, as well as whether women with PCOS require enhanced screening for CE. This study was to assess the incidence of CE among infertile women with PCOS by hysteroscopy combined with histopathology CD138 immunohistochemical staining of endometrium.

**Methods:**

A total of 205 patients in the PCOS group and 4021 patients in the non-PCOS group from July 2017 to August 2022 were included in this retrospective study. After nearest-neighbor 1:4 propensity score matching (PSM), 189 PCOS patients were matched with 697 non-PCOS patients. Basic information was recorded. The CE incidence was compared. The risk factors affecting CE incidence were also analyzed.

**Results:**

No significantly higher CE incidence in infertile women with PCOS were found either in total analysis or after PSM (*P* = 0.969; *P* = 0.697; respectively). Similar results were discovered in the subgroup of Body Mass Index (BMI) (*P* = 0.301; *P* = 0.671; *P* = 0.427; respectively) as well as the four PCOS phenotypes (*P* = 0.157). Intriguingly, the incidence of CE increased as BMI increased in the PCOS group, even though no significant differences were found (*P* = 0.263). Multivariate logistic regression showed that age, infertility duration, infertility type, PCOS, and obesity were not the independent risk factors affecting CE incidence.

**Conclusion:**

The incidence of CE in PCOS patients did not significantly increase compared to non-PCOS patients. Similarly, no significant differences in the incidence of CE were observed among different PCOS phenotypes. The current evidence does not substantiate the need for widespread CE screening among PCOS women, potentially mitigating the undue financial and emotional strain associated with such screenings.

## Background

Polycystic Ovary Syndrome (PCOS), the most prevalent endocrine and metabolic disorder among infertile women, is often associated with chronic low-grade inflammation. Its clinical manifestations encompass hyperandrogenism, insulin resistance, menstrual irregularities, and polycystic ovarian morphology. Studies have linked PCOS with elevated risks of miscarriage and biochemical pregnancy loss [1]. Given that high levels of inflammatory factors could potentially alter the endometrial environment, endometrial dysfunction in women with PCOS warrants careful attention.

Chronic endometritis (CE) is a local inflammation of the endometrium characterized by the infiltration of plasma cells in the endometrial stromal area [2]. CE is known to diminish endometrial receptivity, a crucial factor for successful embryo implantation [3]. The incidence of CE ranges from 2.8 to 56.8% in infertility patients, 14-67.5% in cases of recurrent implantation failure (RIF), and 27-57.8% in instances of recurrent spontaneous abortion (RSA) [4, 5]. Consequently, some clinicians advocate for enhanced CE screening in women at high risk, such as those with endometrial polyps. However, it is still uncertain whether there is a relationship between PCOS and CE, as well as whether women with PCOS require enhanced screening for CE.

Recent studies have demonstrated that CD138 (syndecan-1) can effectively stain plasma cell surfaces, thereby establishing CD138 immunohistochemistry staining as a more reliable diagnostic method for CE, enhancing both its sensitivity and accuracy [6, 7]. A recent study has indicated a higher incidence of CE in infertile patients with PCOS, as diagnosed through hysteroscopy. However, this study utilized conventional diagnostic methods, which rely on the identification of hysteroscopic features such as stromal edema, endometrial micropolyps, or hyperemia for CE diagnosis [8]. Currently, there is a dearth of studies exploring the use of hysteroscopy in conjunction with endometrial CD138 immunohistochemical staining to assess the incidence of CE in infertile women with PCOS. Consequently, this study seeks to investigate the incidence of CE diagnosed by CD138 immunohistochemical staining of endometrial specimens in infertile patients with PCOS, as well as the CE incidence in PCOS women with different clinical phenotypes.

## Materials and methods

This retrospective cohort study was approved by the Ethics Committee of the Sixth Affiliated Hospital of Sun Yat-sen University(2017ZSLYEC-016 S). Infertile patients undergoing hysteroscopic operations from July 2017 to August 2022 were searched. In addition to endometrial polyps, endometrial hyperplasia or other ultrasound abnormalities, patients who underwent hysteroscopy in our hospital usually include patients with unsuccessful artificial insemination, failure of high-quality embryo implantation or unexplained infertility after a simple course of treatment. The participant criteria were as follows: (1) All patients underwent their first hysteroscopy in the proliferative phase and endometrial biopsy during hysteroscopy and the immunohistochemistry staining for CD138 was carried out. (2) No history of cesarean section, intrauterine adhesion, RSA or RIF. (3) No diagnosis of CE before the hysteroscopy in our hospital. (4) No chromosomal abnormalities, congenital uterine malformations, endometrial polyps, endometrial hyperplasia, endometrial tuberculosis, myoma, endometriosis, adenomyosis, cervical intraepithelial lesion, or malignancies of the female reproductive system. (5) No evidence of hydrosalpinx by the diagnosis of ultrasonography or hysterosalpingography, or tubal ligation had not been applied for patients with hydrosalpinx. (6) No evidence of endocrine diseases, except for the patients in the PCOS group (hyperandrogenism, hyperprolactinemia). (7) No rheumatic immune disease.

According to the Rotterdam criteria (2003), two gynecologists at our reproductive center diagnosed patients with PCOS who fulfilled the following criteria: (1) oligo- or anovulation (OA), (2) clinical and/or biochemical signs of hyperandrogenism (HA), and (3) polycystic ovarian morphology (PCOM) with exclusion of other possible causes [9]. HA was defined as total testosterone levels exceeding 0.476 ng/ml or hirsutism with a total score ≥ 8 according to the Ferryman–Gallwey score. OA was defined as a delay of more than 35 days or less than eight spontaneous hemorrhagic episodes per year. PCOM was defined as the presence of 12 or more small follicles measuring 2–9 mm in at least one ovary or an ovarian volume equal to or greater than 10 cm^3^. Patients with PCOS were classified into four phenotype groups as follows: phenotype A (HA + OA + PCOM), phenotype B (HA + OA), phenotype C (HA + PCOM), and phenotype D (OA + PCOM) [10]. According to the recommended criteria for Chinese people, Body Mass Index(BMI) ≥ 28 kg/m^2^, 24–28 kg/m^2^, and <24 kg/m^2^ were categorized into the obese, overweight, and normal weight groups, respectively.

All hysteroscopies and endometrial biopsies were randomly performed by three experienced gynecologists (JP, HZ and ZZ) utilizing KARL STORZ hysteroscopes (Tuttlingen, Germany) to reduce inter-operator bias. The specimens using anti-CD138 antibodies (ZA-0584; ZSGB-BIO, China) were stained under the automatic immunohistochemical staining platform (Benchmark XT, Roche, USA) at our hospital’s pathology department. Whether CD138-positive plasma cells were present in the endometrial tissue samples, as observed on high-power fields (HPF), was counted by an experienced pathologist (YZ). The presence of at least one CD138-positive plasma cell in the endometrial stroma per HPF (400x) was considered indicative of CE, according to previous studies [7, 11].

All datasets were assessed by the Statistical Program for Social Sciences (v 25.0, IBM, IL, USA). The normality of the quantitative data was checked using the Kolmogorov-Smirnov test and expressed as mean ± SD. Quantitative data with normal distribution and homogeneity of variance were compared with Student’s t-test. Quantitative data without normal distribution were compared with the Mann‒Whitney U test. Qualitative data, expressed as frequencies and percentages, were analyzed with the chi-square test or Fisher’s exact test. With the propensity score matching (PSM) extension of SPSS, the propensity score was used to match the following independent variables to balance the influence of confounding factors: age, BMI, infertility duration and type of infertility. A 1:4 match between the PCOS and the non-PCOS group was obtained by the nearest neighbor matching with a caliper width of 0.01. Multivariable logistic regression analysis probed the independent influence of multiple variables in CE, such as age, infertility duration, infertility type, BMI and PCOS. Odds ratio (OR) and 95% confidence interval (CI) were calculated. A value of *P* < 0.05 was considered statistically significant.

## Results

### Basic characteristics

A total of 205 cases were included in the PCOS group, while 4021 cases were included in the non-PCOS group in this study. Table 1 provides the basic characteristics of all cases, as well as the information after nearest-neighbor 1:4 PSM. Significant differences were observed between the two groups for age, BMI, type of infertility (primary or secondary), basal follicle-stimulating hormone (bFSH), basal luteinizing hormone (bLH), anti-Müllerian hormone (AMH), and basal antral follicle count (bAFC) (*P* < 0.05). After PSM, significant differences were only found in the comparisons of PCOS-associated characteristics (bFSH, bLH, AMH and bAFC, *P* < 0.05) and the other baseline characteristics (age, BMI, infertility duration, infertility type) of the two groups were not significantly different (*P* > 0.05). (Tables 1 and 2)

**Table 1:** Clinical characteristics of patients (PCOS and non-PCOS groups)

Items	Before PSM		*P* value	After PSM(1:4)		*P* value
	PCOS group	Non-PCOS group		PCOS group	Non-PCOS group	
Number	205	4021	-	189	697	-
Age, years	30.80 ± 3.76	33.44 ± 8.98	< 0.001	31.05 ± 3.77	31.21 ± 4.26	0.621
BMI (kg/m2)	23.29 ± 3.56	22.04 ± 2.92	< 0.001	22.88 ± 3.24	22.57 ± 2.89	0.229
Infertility duration, years	3.89 ± 2.87	3.87 ± 3.26	0.916	3.82 ± 2.85	3.84 ± 3.01	0.927
Infertility			< 0.001			0.482
Primary infertility %	125/205(60.98%)	1741/4021(43.30%)		109/189(57.67%)	382/697(54.81%)	
Secondary infertility %	80/205(39.02%)	2280/4021(56.70%)		80/189(42.33%)	315/697(45.19%)	

PCOS, polycystic ovary syndrome; BMI, body mass index

**Table 2:** Other PCOS-associated characteristics of patients (PCOS and non-PCOS groups)

Items	Before PSM		*P* value	After PSM(1:4)		*P* value
	PCOS group	Non-PCOS group		PCOS group	Non-PCOS group	
Number	205	4021	-	189	697	-
bFSH (UI/L)	6.18 ± 2.27	7.34 ± 3.72	< 0.001	6.22 ± 2.32	6.93 ± 2.98	0.001
bLH (UI/L)	10.48 ± 7.06	5.80 ± 5.07	< 0.001	10.65 ± 7.20	5.74 ± 4.87	< 0.001
bE2 (pg/ml)	80.67 ± 278.64	74.51 ± 334.51	0.803	82.80 ± 290.34	84.39 ± 393.47	0.960
AMH (ng/ml)	8.32 ± 4.25	3.28 ± 2.80	< 0.001	8.37 ± 4.30	3.83 ± 3.30	< 0.001
bAFC	26.51 ± 7.68	12.40 ± 7.58	< 0.001	17.93 ± 9.74	8.89 ± 6.47	< 0.001

PCOS, polycystic ovary syndrome; bFSH, basal follicle-stimulating hormone; bLH, basal luteinizing hormone; bE2, basal estradiol; AMH, anti-Müllerian hormone; bAFC, basal antral follicle count

### Incidence of CE

**Fig. 1:**
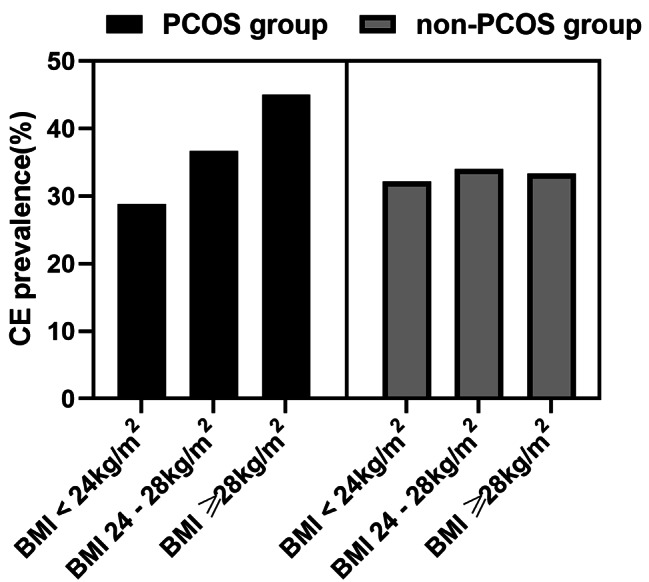
The prevalence of CE among PCOS and non-PCOS women in the subgroup of BMI

**Fig. 2:**
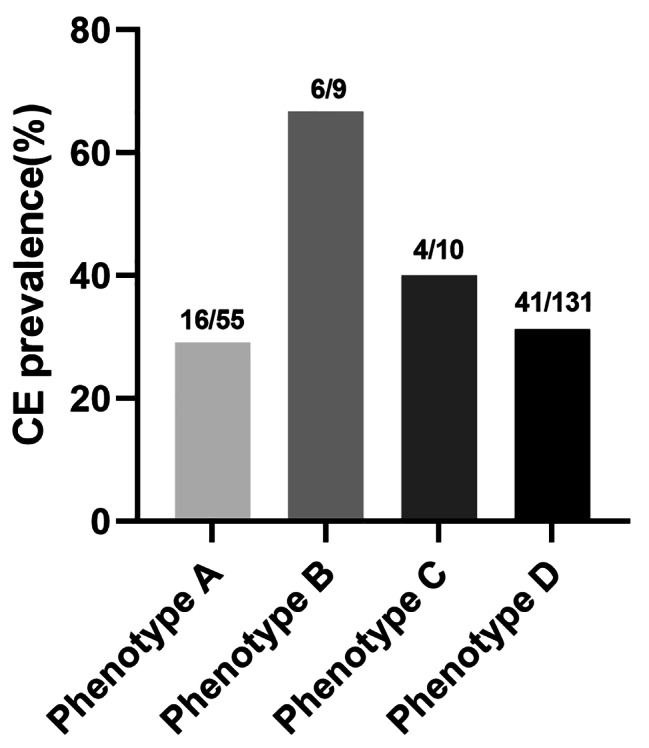
The prevalence of CE among four PCOS phenotypes

There was no significant difference in the incidence of CE among infertile women, irrespective of whether they had PCOS, either in the total analysis or after PSM (*P* = 0.969, OR 1.01, 95% CI: 0.75–1.36; *P* = 0.697, OR 1.07, 95% CI: 0.76–1.51; respectively). Similar results were discovered in the subgroup of BMI ≥ 28 kg/m^2^, BMI 24 -28 kg/m^2^, and BMI<24 kg/m^2^ (*P* = 0.301, OR 1.64, 95% CI: 0.64–4.19; *P* = 0.671, OR 1.13, 95% CI: 0.65–1.95; *P* = 0.427, OR 0.85, 95% CI: 0.58–1.27; respectively, Table 3). Interestingly, the incidence of CE increased as BMI increased in the PCOS group (Fig. 1), even though no significant differences were found (BMI<24 kg/m^2^: 28.80%; BMI 24 -28 kg/m^2^: 36.66%; BMI ≥ 28 kg/m^2^: 45.00%; respectively, *P* = 0.263). The incidences of CE among the four PCOS phenotypes were as follows (Fig. 2): phenotype A group (29.09%, n = 16/55); phenotype B group (66.67%, n = 6/9); phenotype C group (40.00%, n = 4/10); and phenotype D group (31.30%, n = 41/131). However, there was no significant difference in the incidence of CE between these groups (*P* = 0.157). The variables such as age, duration of infertility, type of infertility, presence of PCOS, and obesity did not emerge as independent risk factors for CE, according to the results of the multivariable logistic regression analysis (Table 4).

**Table 3:** Comparison of CE prevalence between PCOS and non-PCOS groups

	PCOS group	Non-PCOS group	*P*	OR (95% CI)
In total	67/205(32.68%)	1309/4021(32.55%)	0.969	1.01(0.75–1.36)
≥ 28 kg/m2	9/20(45.00%)	53/159(33.33%)	0.301	1.64(0.64–4.19)
24–28 kg/m2	22/60(36.66%)	253/745(33.96%)	0.671	1.13(0.65–1.95)
<24 kg/m2	36/125(28.80%)	1003/3117(32.18%)	0.427	0.85(0.58–1.27)
After PSM	59/189(31.22%)	228/697(32.71%)	0.697	1.07(0.76–1.51)

PCOS, polycystic ovary syndrome; OR, odds ratio; CI, confidence interval

**Table 4:** Multivariate logistic regression with CE as a dependent variable

Characteristic	OR	95% CI	*P*
Age	0.99	0.98-1.00	0.104
Infertility duration	1.00	0.98–1.02	0.953
Infertility(Secondary vs. Primary)	1.01	0.88–1.16	0.900
PCOS vs. Non-PCOS	1.02	0.75–1.38	0.905
BMI			
≥ 28 kg/m2	0.96	0.68–1.36	0.824
24–28 kg/m2	0.86	0.62–1.18	0.434
<24 kg/m2	Reference	-	-

PCOS, polycystic ovary syndrome; BMI, body mass index; OR, odds ratio; CI, confidence interval

## Discussion

Accurate diagnosis of CE is crucial because it is frequently asymptomatic and challenging to detect, despite its manifestation as local endometrial inflammation [12]. CD138 is the most specific molecule to identify plasma cells and CD138 immunohistochemical staining has been widely applied in CE diagnosis [13, 14]. It is superior in the detection of CE compared with other methods [15]. The association between PCOS and CE, and the necessity of enhanced CE screening in women with PCOS, remain uncertain. Consequently, we diagnosed CE by hysteroscopy combined with histopathology CD138 immunohistochemical staining of endometrium instead of hysteroscopic findings which included micropolyps, stromal edema and endometrial hyperemia in this study. Our findings indicated that the incidence of CE in PCOS patients did not significantly increase compared to non-PCOS patients. Similarly, no significant differences in the incidence of CE were observed in the subgroup of BMI or the four PCOS phenotypes.

There are several possible explanations for our results. Although both CE and PCOS negatively impact endometrial receptivity, potentially leading to implantation failure [16, 17]. Additionally, circulating and local endometrial inflammatory factors like IL-6, IL-17, and TNF-α are elevated in patients with both PCOS and CE [18]. These suggest that the incidence of CE may be higher in PCOS cases, as previously reported [8]. Nevertheless, we posit that the mechanisms underlying the persistent inflammatory state and diminished reproductive capacity in PCOS and CE may diverge. The existence of microorganisms in the uterus was thought to be the main cause of CE [3]. Microorganisms and endometrial immunity should be seriously considered when dealing with CE [3, 19], inclusive of CE cases with PCOS who had systemic low-grade chronic inflammation. Antibiotic treatment has been reported to be an effective therapy for CE [20]. However, the reduced reproductive potential of PCOS women may be caused by altered oocytes, embryo and endometrial competence, and infertility-related co-morbidities as well as an increased risk of pregnancy complications, regardless of ovulatory status [21].

In PCOS cases, a systemic inflammatory state has been linked to immune responses [22]. Insulin resistance can trigger alterations in cellular immunity, leading to the activation and exacerbation of chronic low-grade inflammation. Notably, the TLR4/IRF-7/NFκB pathway plays a pivotal role in the development of chronic low-grade endometrial inflammation, and metformin has demonstrated the potential to inhibit this pathway, thereby rectifying the state of chronic low-grade inflammation [23]. Furthermore, in PCOS cases, elevated estrogen levels and diminished progesterone levels result in the aberrant expression of sex hormone receptors [22, 24]. Additionally, irregular regulation of enzymatic and metabolic pathways may contribute to endometrial abnormalities [22]. These combined factors, rather than any specific CE, render PCOS cases more susceptible to pregnancy complications, including miscarriage. Importantly, the low-grade chronic inflammation observed in PCOS cases is not attributable to microorganisms. Consequently, the presence of systemic low-grade chronic inflammation in PCOS cases does not necessarily imply a potentially higher incidence of CE. As such, antibiotic treatment is not considered an effective approach for managing low-grade chronic inflammation in PCOS patients. Instead, key strategies for improving chronic inflammation and oxidative stress associated with PCOS involve lifestyle modifications, the use of metformin, and inhibition of androgen synthesis [25]. It may not be beneficial to enhance specific CE screening for all infertile PCOS patients.

In the subgroup analysis of polycystic ovary syndrome (PCOS), we observed that there were more non-obese and non-hyperandrogenic women (phenotype D) in patients with PCOS. This is related to the fact that the phenotypic diversity of PCOS may be affected by ethnic origin, geographic location, and even cultural and social practices [10]. A rising trend of CE incidence was found among obese PCOS cases, even though no significant differences were found. Obesity is associated with a lower pregnancy rate when BMI increases [26]. Obesity promotes low-grade inflammation, as well as hyperandrogenism among PCOS cases [27]. In obese PCOS patients, a higher level of free fatty acid in the endometrium could damage endometrial function [28]. Weigh loss was well established in the restoration of endometrium function [21]. The obesity severity significantly influences the female reproductive system. As mentioned previously, CE may be related to circulating androgens [29]. However, there was no significant difference in the incidence of CE among BMI subgroups and four PCOS phenotypes in this study. We observed an upward trend of CE incidence among the obese cases even though no significant differences were found in this study. This observation may be attributed to the limited sample size within each PCOS subgroup.

Our study boasts several strengths. We employed hysteroscopy in conjunction with endometrial CD138 immunohistochemical staining to assess CE incidence in both PCOS and non-PCOS infertile women, thereby enhancing diagnostic accuracy and objectivity. Age, BMI, infertility duration, and infertility type were meticulously matched between PCOS and non-PCOS groups using PSM to mitigate bias resulting from baseline data imbalances. Furthermore, we conducted CE incidence comparisons among weight subgroups and the four PCOS phenotypes. This retrospective study has two primary limitations. Firstly, different physicians performed hysteroscopy and endometrial biopsy, which could potentially introduce heterogeneity. Standardizing the hysteroscopy procedure at our center and implementing CD138 immunohistochemistry staining for the diagnosis of CE could address this concern. Secondly, the sample size of the PCOS group in our study was limited, highlighting the need for larger sample sizes in future investigations.

## Conclusions

In summary, the incidence of CE in PCOS patients did not exhibit a statistically significant increase compared to non-PCOS patients. Likewise, there were no notable variations in CE incidence across different PCOS phenotypes. The current evidence does not substantiate the need for widespread CE screening among all PCOS women, potentially mitigating the undue financial and emotional strain associated with such screenings. However, an upward trend of CE incidence was found among obese PCOS cases. In the future, prospective studies with a larger sample are needed to investigate the relationship between PCOS and CE, especially for obese PCOS patients.

## Data Availability

The datasets used and/or analyzed during the current study are available from the corresponding author on reasonable request.

## List of Abbreviations

PCOS: Polycystic ovary syndrome
CE: Chronic endometritis
PSM: Propensity score matching
RIF: Recurrent implantation failure
RSA: Recurrent spontaneous abortion
OA: Oligo- or anovulation
HA: Hyperandrogenism
PCOM: Polycystic ovarian morphology
BMI: Body mass index
bFSH: Basal follicle-stimulating hormone
bLH: Basal luteinizing hormone
bE2: Basal estradiol
AMH: Anti-Müllerian hormone
bAFC: Basal antral follicle count
OR: Odds ratio
CI: Confidence interval
